# Optimising desired gain indices to maximise selection response

**DOI:** 10.3389/fpls.2024.1337388

**Published:** 2024-06-24

**Authors:** Reem Joukhadar, Yongjun Li, Rebecca Thistlethwaite, Kerrie L. Forrest, Josquin F. Tibbits, Richard Trethowan, Matthew J. Hayden

**Affiliations:** ^1^ Agriculture Victoria, Centre for AgriBioscience, AgriBio, Bundoora, VIC, Australia; ^2^ School of Life and Environmental Sciences, Plant Breeding Institute, Sydney Institute of Agriculture, The University of Sydney, Narrabri, NSW, Australia; ^3^ School of Life and Environmental Sciences, Plant Breeding Institute, Sydney Institute of Agriculture, The University of Sydney, Cobbitty, NSW, Australia; ^4^ School of Applied Systems Biology, La Trobe University, Bundoora, VIC, Australia

**Keywords:** desired gain indices, genomic prediction, genomic estimated breeding values, selection indices, genomic selection

## Abstract

**Introduction:**

In plant breeding, we often aim to improve multiple traits at once. However, without knowing the economic value of each trait, it is hard to decide which traits to focus on. This is where “desired gain selection indices” come in handy, which can yield optimal gains in each trait based on the breeder’s prioritisation of desired improvements when economic weights are not available. However, they lack the ability to maximise the selection response and determine the correlation between the index and net genetic merit.

**Methods:**

Here, we report the development of an iterative desired gain selection index method that optimises the sampling of the desired gain values to achieve a targeted or a user-specified selection response for multiple traits. This targeted selection response can be constrained or unconstrained for either a subset or all the studied traits.

**Results:**

We tested the method using genomic estimated breeding values (GEBVs) for seven traits in a bread wheat (*Triticum aestivum*) reference breeding population comprising 3,331 lines and achieved prediction accuracies ranging between 0.29 and 0.47 across the seven traits. The indices were validated using 3,005 double haploid lines that were derived from crosses between parents selected from the reference population. We tested three user-specified response scenarios: a constrained equal weight (INDEX1), a constrained yield dominant weight (INDEX2), and an unconstrained weight (INDEX3). Our method achieved an equivalent response to the user-specified selection response when constraining a set of traits, and this response was much better than the response of the traditional desired gain selection indices method without iteration. Interestingly, when using unconstrained weight, our iterative method maximised the selection response and shifted the average GEBVs of the selection candidates towards the desired direction.

**Discussion:**

Our results show that the method is an optimal choice not only when economic weights are unavailable, but also when constraining the selection response is an unfavourable option.

## Introduction

Plant and animal breeding programmes aim to improve multiple commercially valuable traits. Traditional selection methods often overlook the correlation and varying heritabilities among traits, leading to suboptimal selection response. Selecting for traits with conflicting influences can be tricky, as progress in one might harm another (Smith, 1936; Hazel, 1943). Direct selection for traits with antagonistic correlations can lead to unfavourable responses for some of the correlated traits, while synergistic correlations between two or more traits can bias the selection against the remaining traits. Hence, selection indices that account for the architecture and the relationships among different traits are used to simultaneously select for several traits weighted by their importance to the objectives of the breeding programme to avoid unfavourable biases towards or against specific traits (Céron-Rojas and Crossa, 2018).

Selection indices are expected to achieve higher genetic gain compared to independent culling or sequential selection, especially when the targeted traits have variable heritabilities, phenotypic and genotypic correlations, and economic values (Hazel et al., 1994). Selection indices can be applied to phenotypic records, genomic estimated breeding values (GEBVs), or phenotypes and genomic variants (Cerón-Rojas and Crossa, 2020). Despite their utility, selecting the most appropriate indices remains a challenge, requiring a deep understanding of trait genetics and pairwise correlation among the traits (Dekkers et al., 2021). Additionally, the correlation structure among the traits, whether it is due to genetic correlation or environmental correlation, also plays a crucial role (Fernandes et al., 2021). It is important to use a selection index that considers these complexities to mitigate their effects on the selection response (Richardson et al., 2021).

Breeders usually calculate selection indices using the economic values for their traits or through setting a desired gain threshold for each trait (Pesek and Baker, 1969). However, these values may not always be available. Desired gain index methods describe the extent of genetic improvement a breeder intends to achieve for different traits in their germplasm (Pesek and Baker, 1969). The most important advantage of these methods is that they do not require economic weights to be estimated. Desired genetic gain can be determined from a breeder’s knowledge of the genetic merit of their materials and traits, although this may not be possible for all breeding programmes or traits (Céron-Rojas and Crossa, 2018). Alternatively, breeders can sample arbitrary values for the desired gain and choose a sample that shifts all traits towards the desired direction (Li et al., 2017). However, the main problem associated with desired gain selection index methods is that they neither maximise the correlation between the net genetic merit of the individual and the selection index, nor maximise the selection response for different traits (Itoh and Yamada, 1986, 1988).

Here, we report the development of a new iterative method that optimises the choice of the input desired gain (Yamada et al., 1975) to achieve a user-specified selection response for different traits. We demonstrate our method using a large bread wheat population composed of 6,336 lines, of which 3,331 lines were used to develop the selection indices while the remaining 3,005 lines were used as independent selection candidates. Selection indices were developed using the GEBVs for seven traits, namely, grain yield, thousand kernel weight, protein content, screening percentage, and stem rust, leaf rust, and stripe rust disease resistance.

## Materials and methods

### Plant materials and phenotyping

A total of 3,331 wheat lines were used including 36 Australian checks and 2,824 lines developed at the Plant Breeding Institute, Cobbitty, NSW, Australia from different bread wheat germplasm pools and diverse exotic resources including emmer wheat, synthetic wheat, and landraces. More information can be found in Joukhadar et al. (2021a). The population was planted in 30 irrigated field trials between 2014 and 2020 at Narrabri in New South Wales, Cadoux, Merredin, and Geraldton in Western Australia and Horsham in Victoria with population sizes ranging between 195 and 1,956 lines. A subset of approximately 180 lines were selected based on their GEBVs for yield and genetic diversity (Joukhadar et al., 2021b) and replicated across all trials, and different trials shared different numbers of individuals. Materials were sown at two times of sowing (TOS) in randomised complete block designs of two replicates (each replicate as one block) in adjacent blocks. The only exception was Horsham in 2017, where three TOS were used. Details of the 30 trials can be found in **Supplementary Table 1**. Another set of 3,005 double haploid lines were used to validate the selection indices results. These lines were developed from crosses between various parents selected from the reference population of 3,331 individuals.

Seven traits were evaluated, namely, grain yield (YLD), percentage screenings (Screen), protein content (Prot), thousand kernel weight (TKW), and stem rust (Sr), leaf rust (Lr), and stripe rust (Yr) disease resistance. Plots were harvested, and harvested grain weight was subsequently converted to kg/ha as a measure for YLD. Prot was determined using an Infratec TM 1241 Grain Analyser. A total of 400 g of seeds from each plot was screened after 40 machine movements through a screen of 2 mm. The remaining material above the screen was weighed to calculate the screening percentage. One hundred visibly viable seeds (excluding cracked, broken, or diseased seeds) were counted and weighed to determine the 1,000 kernel weight. The three rust diseases were scored for their resistance in a scale from 1 (complete resistance) to 9 (complete susceptibility). The number of trials ranged from 5 for the three rust diseases to 30 for YLD, while the total phenotypic records ranged from 4,566 for Lr and Yr to 11,053 for YLD (**Table 1**). Best linear unbiased estimation (BLUE) values were calculated for each trial independently by fitting a spatial linear mixed model considering the field layout (row and column) and the replications as random effects using ASReml-R (Gilmour et al., 2009). More details can be found in Joukhadar et al. (2021a). BLUE values were used to calculate GEBVs that were used to develop the selection indices.

**Table 1:** Number of trials, total number of phenotypic records, genetic correlation between TOS1 and TOS2 for the studied traits, and prediction accuracy using BayesR.

Trait	Number of trials	Number of records	Genetic correlation	Prediction accuracy
**YLD**	30	11,053	0.89	0.29 (± 0.03)
**TKW**	21	9,872	0.91	0.39 (± 0.04)
**Prot**	17	7,491	0.98	0.32 (± 0.03)
**Screen**	23	8,557	0.92	0.30 (± 0.03)
**Sr**	5	4,633	.	0.40 (± 0.04)
**Yr**	5	4,566	.	0.47 (± 0.05)
**Lr**	5	4,566	.	0.42 (± 0.04)

Prediction accuracy columns show the average of the 100 cross-validation replicates and the value between the brackets represent the standard deviation of the average accuracies. Lr, leaf rust; Sr, stem rust; Yr, yellow rust; Prot, protein content; Screen, screening percentage; TKW, thousand kernel weight; and YLD, grain yield. Genetic correlation was not calculated for the rust diseases because they were only scored in TOS1.

The reference population of 3,331 individuals was genotyped with the 90K Infinium SNP array (Wang et al., 2014). SNPs were filtered to keep only the one with call rate > 60% and minor allele frequency (MAF) > 5%. Missing genotypes within the 90K SNPs were imputed using LinkImpute software (Money et al., 2015) and the previous studies showed that the accuracy of the *in silico* cross-validation was larger than 0.99 (Joukhadar et al., 2020). The validation population of 3,005 individuals was genotyped with 40K wheat and barley Infinium SNP array (Keeble-Gagnère et al., 2021). For all materials, a single plant was genotyped given that all individuals were even double haploid or fixed lines. Both the reference population genotyped with 90K and the validation population genotyped with 40K Infinium SNP arrays were imputed to exome capture level (He et al., 2019). The imputation from low density to high density was previously reported to have an accuracy of 92.4% (Joukhadar et al., 2021a). A total of 218,092 were common across the reference and the validation imputed SNP sets, which were used for subsequent analyses (Joukhadar and Daetwyler, 2022).

### Genetic correlation and genomic estimated breeding values

Trait variances and narrow-sense heritability (SNP based heritability) was calculated using the univariate restricted maximum likelihood (REML) analysis implemented in MTG2 software (Lee and Van der Werf, 2016) by fitting the phenotypic records as well as the genomic relatedness matrix. The genetic covariances and genetic correlations between each pair of traits were calculated using bivariate REML analysis implemented in the same software. For both analyses, the trials were fitted as a covariate. The genetic correlation analysis was performed with two aims: (1) to calculate the genetic correlation between the two times of sowing (TOS1 and TOS2) to assess the level of genotype by environment interaction between both TOSs; and (2) to calculate the genetic covariances among traits, which is required to develop the selection indices.

GEBVs were calculated using the BayesR software, previously described in Breen et al. (2022). BayesR models SNP effects from a mixture of four normal distributions (Erbe et al., 2012). The aim is to simultaneously assign the SNPs with large, medium, small, and no effect. Marker effects were sampled from one of the following four normal distributions: N(0, 0 $\sigma_{g}^{2}$ ) zero effect, N(0, 0.0001 $\sigma_{g}^{2}$ ) small effect, N(0, 0.001 $\sigma_{g}^{2}$ ) medium effect, and N(0, 0.01 $\sigma_{g}^{2}$ ) large effect, where $\sigma_{g}^{2}$ is the additive genetic variance. The genomic relatedness matrix was calculated following VanRaden (2008). The prior proportions of loci attributed to each marker effect distribution were 0.94, 0.049, 0.01, and 0.001, respectively. A total of 50,000 iterations for the BayesR analysis were used, of which the first half was considered as burn in. For cross-validation, half of the phenotypic records for each trait were randomly selected as a reference to predict the other half, which was considered the validation set. Prediction accuracy was calculated as the correlation coefficient between the GEBVs and the actual phenotypes in the validation set. The cross-validation strategy was repeated 100 times, wherein the population was randomly partitioned into reference and validation sets in each replicate. Prediction accuracies for each trait were averaged across the 100 replicates, and the standard deviation of the average accuracies was subsequently calculated.

### Desired gain index

Selection indices were calculated for the following traits: grain yield, thousand kernel weight, screening percentage, protein content, and leaf, stem, and yellow rust disease resistance. We used the desired trait gain index method described in Yamada et al. (1975). The method uses the following equation:


(1) $$b=P^{-1}G{\left(GP^{-1}G\right)}^{-1}d$$

where ***b*** is the final desired gain-based index; ***P*** is the phenotypic variance–covariance matrix (in our case, the correlation between GEBVs); ***G*** is the genetic variance–covariance matrix; and ***d*** is a vector of the desired gains. Variances in ***G*** were calculated using the previous univariate REML analysis for each trait, while the covariances were calculated by applying the bivariate REML model to analyse each pair of traits.

Yamada et al. (1975) suggested choosing arbitrarily values for ***d*** until the averages for all traits in the selected lines were shifted towards the desired direction (e.g., increasing yield and reducing screening percentage). Instead of choosing arbitrary values for ***d***, we developed a new strategy that optimises the choice of ***d*** to calculate an optimal index (***b***) that can achieve a user-specific level of improvement (selection response) for each trait. The user-specified selection response (***dg***) is defined as the number of standard deviations (positive or negative) the average phenotypes of the selected lines can be shifted from the average of the whole population for each trait. The method samples values for ***d*** within an iterative process and selects the sample that develops an index that best matches the desired selection response. For each iteration, the method calculates ***b*** and use it to calculate the genetic gain ***g*** of the sampled ***d***. Then, ***g*** is used to calculate the goodness of fit for the sampled ***d*** for each trait with the following equation:


(2) $$\text{gof}=\frac{\log_{base}\left(\frac{base^{2}}{\left|g\right|}\right)}{\frac{base^{2}}{\left|g\right|}}$$

where “base” is the base of the logarithm that can be calculated with the following equation: $\text{base}=\sqrt{\left|e\times dg\right|}$ , in which *e* is the Euler’s number. Specifically, the base determines the scaling of the logarithmic transformation applied to the ratio of ***g*** to ***dg***. Therefore, under the proposed equation, ***gof*** gets maximised (***gofMAX***) when ***g*** = ***dg***, which means that we achieved the desired selection response in the selection candidates. The penalty of the sampled d for all traits (θ) is given by the following equation:


(3) $$\text{θ}=\sum_{i}^{n}\frac{{\text{gofMAX}}_{i}-{\text{gof}}_{i}}{{\text{gofMAX}}_{i}}$$

where *n* is the number of traits. Θ serves as a measure of deviation between the observed and desired selection responses across traits, guiding the iterative optimisation process. A lower value of θ indicates better alignment between **g** and ***dg***. The value of θ from each iteration is compared to the sample of the previous iteration and is accepted if the selection response improves, i.e., getting a smaller θ. The sampling mean for each trait in each iteration is updated from the most recently accepted sampled weights. The analysis was run for 1,000 iterations.

We used the GEBVs for the phenotyped reference population of 3,331 lines to develop genotypic and phenotypic covariance matrices. The calculated covariance matrices calculated on the reference population were then applied to the GEBVs of the 3,005 double haploid selection candidates. From the selection index ranked lines, a total of 100 lines were selected. Selection response for the selected lines was expressed as the number of standard deviations their average differed from the average of the whole population for each trait. Three different indices were calculated. The user-specified selection response was first set to +0.5 standard deviations for yield, TKW, and protein, and −0.5 standard deviations for screening the three rust diseases (INDEX1) to ensure equal weights for all traits. Another yield dominant user-specified selection response was used with +2 standard deviations for yield, +0.5 standard deviation for TKW and protein, and −0.5 standard deviation for the three rust diseases (INDEX2). A third index was calculated with high user-specified selection responses at +4 standard deviations for yield, TKW, and protein, and −4 standard deviations for screening the three rust diseases (INDEX3) to maximise the potential selection response for all traits. Each index was run for 20 replicates and the correlations among replicates were averaged to ensure that the different replicates produced comparable solutions.

## Results

The analysis revealed high genetic correlations among all traits, indicating limited genotype by environment interactions for TOS1 and TOS2. Notably, the smallest correlation observed was 0.89 for grain yield while the largest correlation was equal to 0.98 for protein percentage (**Table 1**). This analysis was not possible for the three rust diseases given that the disease resistances were scored only in optimal sowing time (TOS1). Additionally, the BayesR model demonstrated varying levels of prediction accuracy for the studied traits, ranging from medium to low, with values between 0.29 for YLD and 0.47 for Yr (**Table 1**). Particularly noteworthy were the higher prediction accuracies observed for resistances to the three rust diseases, averaging at 0.43, compared to an average of 0.33 for the remaining four traits.

The narrow-sense heritability and genetic correlations among the studied traits were summarised in **Table 2**. Narrow-sense heritability values varied across the traits, ranging from 0.21 for YLD to 0.59 for Yr. Additionally, the examination of genetic correlations revealed generally low values among the seven traits, spanning from −0.21 to 0.28, underscoring the relative independence of these traits in terms of their genetic basis. However, a striking exception to this pattern was observed in the strong negative correlation of −0.61 between TKW and screening percentage, which is expected given that small, low weight seeds are the ones that pass through the screen. Overall, these findings contribute to a deeper understanding of the genetic relationships and heritability of the studied traits, providing valuable insights for future breeding efforts aimed at improving crop performance and resilience.

**Table 2:** Narrow-sense heritability (diagonal) and genetic correlations (off diagonal) between the studied traits.

	YLD	TKW	Prot	Screen	SR	YR	LR
YLD	0.21 (± 0.01)	−0.21 (± 0.07)	−0.03 (± 0.09)	0.10 (± 0.09)	0.16 (± 0.09)	0.12 (± 0.08)	0.16 (± 0.09)
TKW	−0.21 (± 0.07)	0.50 (± 0.01)	−0.01 (± 0.07)	−0.61 (± 0.05)	−0.05 (± 0.07)	0.14 (± 0.06)	0.28 (± 0.07)
Prot	−0.03 (± 0.09)	−0.01 (± 0.07)	0.30 (± 0.02)	0.15 (± 0.08)	−0.10 (± 0.07)	−0.15 (± 0.07)	0.13 (± 0.08)
Screen	0.10 (± 0.09)	−0.61 (± 0.05)	0.15 (± 0.08)	0.49 (± 0.01)	−0.06 (± 0.08)	−0.14 (± 0.07)	−0.18 (± 0.08)
SR	0.16 (± 0.09)	−0.05 (± 0.07)	−0.10 (± 0.07)	−0.06 (± 0.08)	0.35 (± 0.02)	−0.07 (± 0.06)	0.11 (± 0.08)
YR	0.12 (± 0.08)	0.14 (± 0.06)	−0.15 (± 0.07)	−0.14 (± 0.07)	−0.07 (± 0.06)	0.59 (± 0.01)	0.28 (± 0.07)
LR	0.16 (± 0.09)	0.28 (± 0.07)	0.13 (± 0.08)	−0.18 (± 0.08)	0.11 (± 0.08)	0.28 (± 0.07)	0.34 (± 0.02)

Values between brackets represent the standard errors of the estimations. Lr, leaf rust; Sr, stem rust; Yr, yellow rust; Prot, protein content; Screen, screening percentage; TKW, thousand kernel weight; and YLD, grain yield.

Iterative index analysis (Equations 2, 3), run using the parameters of the three tested indices, identified a subset of samples with average GEBVs that were all shifted towards the desired direction of increased yield, TKW, and protein; reduced screenings; and low rust disease resistance scores (**Tables 3**–**5**). The iterative method showed better performance compared to the standard desired genetic gain method (Yamada et al., 1975) with no iterations. For INDEX1 (equal weight for all traits), the absolute standard deviation of the response ranged between 0.46 and 1.02 with an average of 0.68, which was very close to the targeted user-specified response of 0.5 for all traits. On the other hand, without iteration, the absolute standard deviation of the response ranged between 0.12 and 1.69. The iterative method also successfully developed an index when the targeted response was biased towards a subset of trait(s). INDEX2 (the yield dominant index) had a higher weight for yield and selected lines with the iterative method had, on average, standard deviations 1.71 higher than the whole population. The average of the remaining traits was higher at approximately 0.5 standard deviations, which equalled the user-specified targeted response (**Table 4**). However, protein content was negatively selected when the index was used without iterations, indicating the superiority of the new method.

**Table 3:** Selection response from INDEX1.

Trait	Sampled desired gain (*d*)	Index (*b*)	Targeted response (*dg*)	Response NoIteration	Response SD Ref	Response SD Cand
YLD	1.06	16.39	0.5	0.50	0.67	0.83
TKW	3.37	1.34	0.5	1.69	0.57	1.02
Prot	0.72	1.26	0.5	0.48	0.87	0.66
Screen	−1.22	0.15	−0.5	−0.12	−0.43	−0.46
SR	−0.55	−2.91	−0.5	−0.31	−0.55	−0.56
LR	0.03	−1.67	−0.5	−0.95	−0.58	−0.61
YR	−0.60	−1.14	−0.5	−0.29	−0.53	−0.62

The sampled desired gain (*d*), final index (*b*), user-specified targeted selection response (*dg*), and number of standard deviations for the genetic gain of the selected lines [NoIteration, using the standard desired gain index method of Yamada et al. (1975), our method with the reference, Ref, and candidate, Cand, populations] using INDEX1 (equal weights for all traits). Lr, leaf rust; Sr, stem rust; Yr, yellow rust; Prot, protein content; Screen, screening percentage; TKW, thousand kernel weight; and YLD, grain yield; SD Ref, standard deviation reference population; and SD Cand, standard deviation selection candidates.

**Table 4:** Selection response from INDEX2.

Trait	Sampled desired gain (*d*)	Index (*b*)	Targeted response (*dg*)	Response NoIteration	Response SD Ref	Response SD Cand
YLD	1.61	19.70	2	2.01	1.33	1.71
TKW	−0.28	0.58	0.5	0.74	0.33	0.46
Prot	0.48	1.27	0.5	−0.35	0.44	0.51
Screen	0.01	0.04	−0.5	−0.08	−0.45	−0.50
SR	0.70	1.40	−0.5	−0.21	−0.46	−0.54
LR	0.53	−0.01	−0.5	0.13	−0.44	−0.54
YR	0.65	−0.61	−0.5	−0.45	−0.42	−0.54

The sampled desired gain (*d*), final index (*b*), user-specified targeted selection response (*dg*), and number of standard deviations for the genetic gain of the selected lines [NoIteration, using the standard desired gain index method of Yamada et al. (1975), our method with the reference, Ref, and candidate, Cand, populations] using INDEX2 (yield dominant). Lr, leaf rust; Sr, stem rust; Yr, yellow rust; Prot, protein content; Screen, screening percentage; TKW, thousand kernel weight; and YLD, grain yield; SD Ref, standard deviation reference population; and SD Cand, standard deviation selection candidates.

**Table 5:** Selection response from INDEX3.

Trait	Sampled desired gain (*d*)	Index (*b*)	Targeted response (*dg*)	Response NoIteration	Response SD Ref	Response SD Cand
YLD	0.24	5.47	4	0.50	0.78	1.05
TKW	1.35	0.28	4	1.69	0.53	0.64
Prot	0.57	1.33	4	0.48	0.73	1.10
Screen	−3.05	−0.40	−4	−0.12	−0.49	−1.24
SR	−0.62	−2.53	−4	−0.31	−0.50	−1.09
LR	−0.75	−2.35	−4	−0.95	−0.65	−1.06
YR	−0.78	−0.74	−4	−0.29	−0.60	−1.11

The sampled desired gain (*d*), final index (*b*), user-specified targeted selection response (*dg*), and number of standard deviations for the genetic gain of the selected lines [NoIteration, using the standard desired gain index method of Yamada et al. (1975), our method with the reference, Ref, and candidate, Cand, populations] using INDEX3 (maximum gain). Lr, leaf rust; Sr, stem rust; Yr, yellow rust; Prot, protein content; Screen, screening percentage; TKW, thousand kernel weight; and YLD, grain yield; SD Ref, standard deviation reference population; and SD Cand, standard deviation selection candidates.

**Figure 1:**
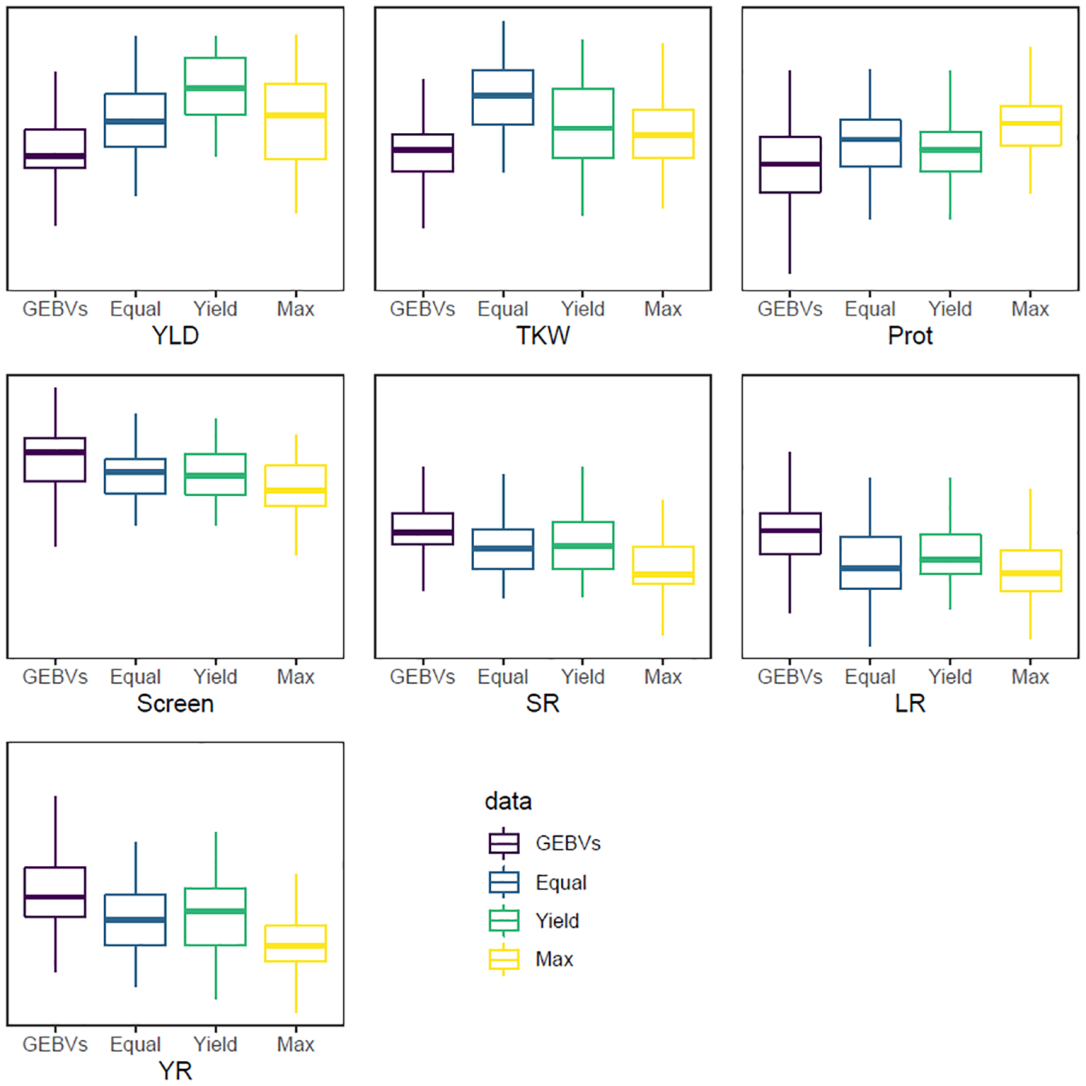
Boxplots showing the distribution of the GEBV values (*y*-axis) for the seven selected traits. GEBVs: the whole selection candidates’ population; Equal: INDEX1 that has equal weight for all traits; Yield: for INDEX2 that has higher weight for grain yield; and Max: for INDEX3 that maximises the response for all traits. For YLD, TKW, and Prot, the aim is to increase the trait while the aim for Screen, SR, LR, and YR is to reduce the trait.

The last index, INDEX3, had a targeted response of 4 standard deviations across all traits, which was impossible to achieve given that only 100 lines were selected from a population of 3,005 individuals (~3.3%). This index was designed to maximise the genetic gain for all traits in the selected materials as, theoretically, only 0.05% of the whole population could be above or below 4 standard deviations for a single trait. The average GEBVs of the selected materials had an absolute average of 1.04 standard deviations from the average of the whole population across the seven traits, which ranged from 0.64 for TKW to 1.24 for screenings percentage (**Table 5**). Calculating this index without iterations resulted in the same answer as INDEX1 given that they both have equal weights across the traits and *d* is a scaler in Equation 1. Therefore, the ranking of individuals will be the same for both INDEX1 and INDEX3. **Figure 1** shows the distribution of YLD for the selection candidate whole population as well as the selected lines using the three indices (INDEX1, INDEX2, and INDEX3).

The method was validated using 3,005 double haploid lines, which were independent from the reference set that was used to calculate the phenotypic and genotypic covariance matrices. The selection response for the independent validation set was very similar but slightly higher than that calculated on the reference population (**Tables 3**–**5**). In other words, the average phenotypes for the selection candidates in both the reference and the validation sets had an equivalent number of standard deviations above (for YLD, TKW, and protein) or below (for screening and the three rust diseases) the average of the whole reference or validation populations, respectively. Running 20 replicates for each analysis showed that the iterative method produced indices with high repeatability. The average correlation coefficients over all replicates for the three indices were 0.95, indicating robust divergence after 1,000 iterations as well as consistently highly optimised weights for different traits.

## Discussion

We utilised a large population of Australian bread wheat cultivars and breeding lines that were phenotyped in diverse environments across the Australian wheatbelt, stretching from Narrabri in New South Wales to Geraldton in Western Australia. The materials were planted at both optimal (TOS1) and late (TOS2) times of sowing and irrigated to avoid drought stress so that heat tolerance during anthesis and grain filling periods could be assessed (Thistlethwaite et al., 2020).

To develop robust selection indices for different traits, it is important to quantify the level of genotype by environment interaction between the optimal and the late sowing trials by calculating their genetic correlation. The high genetic correlation observed implied that the ranking of the lines had minimal change between the two sowing times for all traits. This is essential to decide whether a trait will need a single weight regardless of heat treatment or if two different weights will be needed, one for each treatment. All traits showed high correlations between both TOSs of which protein content had almost no genotype by environment interaction with a genetic correlation between TOS1 and TOS2 of 0.98 (**Table 1**). This was not unexpected given that protein content is affected more by nitrogen availability in the soil than weather (McDonald, 1992) and TOS1 and TOS2 were planted in adjacent fields in each environment and subjected to the same management practises. These results indicated that TOS1 and TOS2 did not need different weights when developing the selection indices and could be considered as a single trait.

The prediction accuracy for resistance to the three rust diseases was comparable to accuracies reported in previous studies that investigated diverse wheat germplasms (Daetwyler et al., 2014; Juliana et al., 2017). He et al. (2019) used a subset of the data used in the present study that included 10 trials conducted at Narrabri, New South Wales between 2013 and 2017. They reported comparable prediction accuracies to our results for grain yield and screening percentage for models that did not fit genotype by environment interactions. However, their accuracies for protein content and TKW were much higher compared to our results. The latter could be a result of the inclusion of field trials with more diverse climates and soil types in the reference population used in our study. He et al. (2019) also showed that prediction accuracy was further improved for protein content and grain yield when fitting the genotype by environment interaction in the model. However, our research focussed on improving the development of indices to maximise the selection response regardless of the statistical model applied for genomic prediction.

The seven studied traits showed low to medium narrow-sense heritability (**Table 2**). Daetwyler et al. (2014) reported comparable heritability values to our estimates for resistance to the three rust diseases. Various studies have reported comparable heritability values for the remaining traits, especially low heritability for YLD which is often highly influenced by environmental variability (Babar et al., 2007; Heffner et al., 2011; He et al., 2019; Watson et al., 2019). The genetic correlations among different traits were generally low and in most cases the value was smaller or equivalent to two standard errors, except for the negative correlation between TKW and screening percentage of −0.61 (**Table 3**). A negative correlation is expected given that a higher screening percentage implies smaller seeds. The remaining traits showed low genetic correlations between −0.21 and 0.28, indicating that selection applied to a given trait will not significantly affect another trait.

Previously developed unconstrained linear phenotypic or genomic selection indices do not have control over the direction of the selection response; i.e., whether genetic gain is increased or decreased (Smith, 1936; Dekkers, 2007; Togashi et al., 2011; Cerón-Rojas and Crossa, 2019). For this reason, attempts have been made to constrain the genetic gain for a subset of traits. Kempthorne and Nordskog (1959) developed a method to prevent changing of the genetic gain for a subset of traits (in other words, the gain is equal to zero), while others generalised this method to allow the setting of a predetermined level of genetic gain for a subset of traits (Mallard, 1972; Harville, 1975; Tallis, 1985). These methods improved the choice of selection candidates with better shifts in genetic gain in the desired direction. However, all these methods required an economic weight for each trait to be predefined, information that may not be always available for all traits or breeding programmes.

While the advantage of the desired gain indices is that economic weights are not required, their main disadvantage is inability to maximise the selection response (Itoh and Yamada, 1986, 1988). This results because of the subjective choice of the desired gain values that are usually approximated using the breeder’s knowledge of the traits or arbitrarily sampled to achieve a proper selection response (Céron-Rojas and Crossa, 2018). Cerón-Rojas and Crossa (2020) argued that the constrained linear phenotypic selection index is similar in principle to the desired gain indices with the advantage of maximising the selection response as well as the correlation between the selection index and the net genetic merit of each individual. Our iterative method optimises the choice of the desired gain to achieve a user-specified selection response for the targeted traits. Our results show that our method can efficiently move the genetic gain in the desired direction regardless of whether the breeding objective requires some traits to be constrained (INDEX1 and INDEX2) or unconstrained (INDEX3). Our method optimises the selection response not only when the economic weights are unavailable, but also when constraining a subset of traits is not the ideal option.

While our method succeeded in maximising the selection response for all traits in the desired direction, it is still not possible to determine if the method maximises the correlation between the selection index and net genetic merit. This is because the covariance between both is undefined given that it depends on the estimation of the economic weight (Cerón-Rojas and Crossa, 2020); thus, this parameter cannot be theoretically assessed using our method. However, as our validation population of 3,005 lines was developed from crosses between a selected subset of parents from the reference population of 3,331 individuals, the former materials can be considered as the next generation in a continuous breeding programme that was not included in the reference population. Hence, this population can empirically help assess the unobserved genetic merit. Similar results were previously obtained using unconstrained and constrained linear phenotypic or genomic selection indices using real data with two breeding cycles or simulated data with six cycles derived from the reference population (Cerón-Rojas and Crossa, 2019, 2020).

Our new approach holds significant practical implications for enhancing breeding efficiency and accelerating genetic gain in improving breeding programmes. Our method aids breeders in the selection of superior cultivars with broad adaptability simultaneously for multiple traits without biasing the selection towards or against a specific trait. Additionally, our iterative method for optimising selection responses, particularly in scenarios where economic weights are unavailable or when constrained selection is not ideal, offers a flexible and efficient approach to breeding programme management. Overall, our study contributes to advancing breeding methodologies tailored to modern breeding objectives, ultimately facilitating the development of improved varieties with enhanced yield potential, stress resilience, and end-use quality.

## Conclusion

We developed a new method that extends the application of desired trait gain selection indices to maximise the selection response for multiple constrained or unconstrained traits. We showed that our method shifts the selection response in non-reference individuals in the targeted direction to increase or decrease the trait average in the selection candidates. We demonstrated that our method has the power to maximise the genetic gain when using unconstrained weights and to achieve the targeted selection response set by the breeder for different traits in the appropriate direction. However, more empirical testing is required using multi-breeding cycle data to ensure that the calculated indices are sufficiently powerful to maximise the correlation between the index and the net genetic merit.

## Data availability statement

The raw data supporting the conclusions of this article will be made available by the authors, without undue reservation.

## Author contributions

RJ: Writing – original draft, Visualization, Validation, Software, Resources, Project administration, Methodology, Investigation, Funding acquisition, Formal analysis, Data curation, Conceptualization. YL: Writing – review & editing, Software, Resources, Methodology, Investigation, Formal analysis, Conceptualization. RTh: Writing – review & editing, Resources, Funding acquisition, Data curation. KF: Writing – review & editing, Resources, Data curation. JT: Writing – review & editing, Resources, Project administration, Data curation. RTr: Writing – review & editing, Supervision, Resources, Project administration, Funding acquisition, Data curation, Conceptualization. MH: Writing – review & editing, Supervision, Resources, Project administration, Funding acquisition, Conceptualization.

## Supplementary material

Supplementary Table 1 Summary of the field trials, number of phenotyped individuals, and traits. Trail names were recorded as (Location_year_TimeOfSowing). NoGeno: Number of Genotypes; Prot, protein%; Screen, screening%; TKW, thousand kernel weight (g); YLD, yield (t/ha); Lr, leaf rust; Sr, stem rust; Yr, stripe rust.
